# Research progress of mesenchymal stem cells/stromal cells and their derivatives against cell senescence in the treatment of osteoarthritis

**DOI:** 10.1080/07853890.2026.2627667

**Published:** 2026-04-01

**Authors:** Daihua Deng, Yinlan Wu, Tong Wu, Deying Huang, Xiuping Liang, Chunyu Tan, Yanhong Li, Yi Liu

**Affiliations:** ^a^ Department of Rheumatology and Immunology, Laboratory of Rheumatology and Immunology, West China Hospital, Sichuan University, Chengdu, China; ^b^ Department of Rheumatology, Mianyang Central Hospital, School of Medicine, University of Electronic Science and Technology of China, Mianyang, China

**Keywords:** Mesenchymal stem cells/stromal cells, anti-cell senescence, osteoarthritis, mesenchymal stem cell derivative

## Abstract

**Background:**

Cellular senescence plays a critical role in the pathogenesis and progression of osteoarthritis (OA), contributing to articular cartilage degradation, chronic inflammation, and joint function impairment. Mesenchymal stem cells/stromal cells (MSCs) and their derivatives have emerged as potential targets for novel therapeutic strategies against cell senescence in OA, as they exert anti-aging effects in repairing damaged cartilage through multiple mechanisms—including regulating age-related signaling pathways, reducing the secretion of pro-inflammatory cytokines, and improving mitochondrial function.

**Discussion:**

In recent years, a growing body of in-depth studies has focused on various types of MSCsand their derivatives, revealing subtle differences in their anti-cell senescence pathways and therapeutic effects. This review summarizes the latest cutting-edge research, systematically exploring the multi-dimensional roles of these MSCs and their derivatives in ameliorating senescent cell-related pathology in OA, and highlighting their potential clinical application value.

**Conclusions:**

Despite the promising anti-aging effects of MSCs and their derivatives in OA treatment, further molecular-level mechanistic studies and large-scale clinical trials are required to verify the efficacy and safety of therapies based on their anti-cell senescence properties. These efforts are expected to provide new breakthroughs in the clinical management of OA, offering more effective treatment options for patients.

## 1. Introduction

Osteoarthritis (OA) is a chronic joint disease primarily characterized by the degeneration of articular cartilage, the formation of osteophytes at the joint margins, subchondral bone sclerosis and chronic synovial inflammation [1]. It is predominantly a degenerative disease commonly affecting weight-bearing joints such as the knees and hips [2]. Currently, OA is the most common joint disease worldwide, with an estimated 595 million people affected globally. The incidence is higher in women and the elderly population. With the ageing population, the number of OA cases continues to rise [3]. The most apparent clinical symptoms in OA patients include joint pain, stiffness, swelling and limited mobility. These symptoms progress slowly and insidiously over time, eventually leading to a gradual loss of joint function, severely impacting daily activities and work capabilities [2]. Chronic joint pain and functional impairment not only cause physical suffering but also often lead to psychological issues such as depression and anxiety, increasing the social and economic burden of healthcare [4]. Therefore, effective and proactive treatment of OA is of paramount importance.

Current treatments for OA primarily include pharmacological therapy, physical therapy and surgical intervention [5]. Pharmacological treatments often involve the use of nonsteroidal anti-inflammatory drugs (NSAIDs) and analgesics to alleviate pain and inflammation. It is important to note that these medications may cause gastrointestinal discomfort and cardiovascular risks [6]. Physical therapy includes joint function exercises and physical modalities that help to improve joint mobility and reduce pain [7]. Surgical interventions, such as joint replacement surgery, are typically reserved for severe OA patients and can significantly improve joint function [8]. Nevertheless, only a portion of OA patients are eligible for surgical treatment [9]. Therefore, current treatment modalities only focus on relieving symptoms but fail to control disease progression or repair damaged cartilage [10].

In recent years, mesenchymal stem cells/stromal cells (MSCs) [11] and their derivatives have emerged as a novel therapeutic approach which have been widely explored in the research and clinical treatment of OA [12]. MSCs possess the potential of self-renewal and multipotent differentiation, allowing them to differentiate into chondrocytes in specific environments, thereby renewing and repairing damaged or aged tissues [13]. Some studies have found that MSCs and their derivatives not only exhibit good cartilage regeneration abilities in experimental models but also mitigate OA symptoms by modulating immune responses and exerting anti-inflammatory effects [14,15]. Notably, they showed potential therapeutic effects in anti-senescence pathways, which could delay the ageing process of chondrocytes [16], thereby improving the pathological state of OA. To some extent, they can even mitigate disease progression by regulating tissue-cell pathological changes.

Therefore, this review aims to elucidate and summarize recent advances in the research on MSCs and their derivatives in treating OA through anti-senescence pathways, analyzing their potential scientific research and clinical value. By summarizing cutting-edge research, we hope to provide insights and references for future related studies and promote the application and development of this emerging therapy in OA treatment.

## 2. Discussion

### 2.1. Pathogenesis and progression of OA

During the onset and progression of OA, genetic factors, mechanical stress and inflammation collectively contribute to the degradation of articular cartilage [17]. In the early stages, chondrocytes are stimulated by mechanical stress and inflammatory factors, leading to the excessive production of matrix metalloproteinases (MMPs) that degrade the cartilage matrix and results in mild pain and stiffness [18]. As the disease progresses, chondrocytes experience proliferation defectiveness and apoptosis, followed by the intensification of cartilage matrix damage and occurrence of subchondral bone sclerosis. Thereby, causing patients to experience significant joint pain, swelling and limited mobility [19–21]. In the late stages, the cartilage is completely degraded and disappeared, together with narrowed joint space and the formation of osteophytes that eventually leading to severe functional impairment and persistent pain [22].

It is noteworthy that cellular senescence plays a critical role in the onset and progression of OA. Senescent chondrocytes secrete inflammatory factors and degradative enzymes which accelerates cartilage degradation [23]. Therefore, therapeutic strategies targeting cellular senescence hold promise for fundamental improvement of the pathological state of OA and delaying or halting disease progression. Cellular senescence refers to the permanent growth arrest state that cells enter after a certain number of divisions or in response to various stressors, results in losing their proliferative capacity while retaining metabolic activity [24]. The mechanisms of senescence mainly include DNA damage response, telomere shortening, oxidative stress and inflammatory responses [25,26]. After senescence, the ability to express and secretory various extracellular regulators was defined as senescence-associated secretory phenotype (SASP) [27]. In OA, chondrocytes, synovial cells and subchondral bone cells exhibit this significant senescence characteristics [16,28]. Moreover, their roles vary in different disease stages to drive the development and deterioration of OA: in the early stage, senescent cells secrete pro-inflammatory factors and MMPs that degrade joint cartilage [29]; in the middle stage, senescent cells increase joint tissue inflammation and accelerate cartilage degradation [30]; in the late stage, senescent cells accumulate in the joint cavity, forming the SASP and further worsening the tissue microenvironment to promote disease progression [31]. Therefore, anti-senescence therapeutic strategies are crucial for alleviating and improving OA. By clearing senescent cells, inhibiting SASP and promoting cell regeneration, inflammation can be effectively controlled and damaged tissues can be repaired [32]. Meanwhile, MSCs and their derivatives show tremendous potential in these aspects, as they can secrete anti-inflammatory factors and promote tissue repair, delaying or reversing the pathological process of OA [33]. Therefore, in-depth research on the role of MSCs and their derivatives in combating cellular senescence is of great significance in developing new and effective treatments for OA.

### 2.2. Mesenchymal stem cell treatment

MSCs are a class of cells with the potential for self-renewal and multipotent differentiation and can be categorized into embryonic stem cells and adult stem cells based on their source [34]. Adult MSCs can be further classified according to the tissue of origin, such as bone marrow-derived mesenchymal stem cells/stromal cells (BM-MSCs), adipose-derived mesenchymal stem cells/stromal cells (AD-MSCs) and umbilical cord mesenchymal stem cells/stromal cells (UC-MSCs) [35]. With the development of reprogramming technology and developmental biology, induced pluripotent stem cell-derived mesenchymal stem cells/stromal cells (iMSCs) [36], human embryonic stem cell-derived mesenchymal stem cells/stromal cells (EMSCs) [37] and dental pulp-derived stem cells (DPSCs) [38,39] have also shown significant potential for cartilage repair. In recent years, stem cell therapy has demonstrated stability, safety and substential therapeutic effects in the clinical application for various diseases, including heart disease, neurological disorders and immune diseases [40].

Besides the stem cells themselves, stem cell derivatives like exosomes (Exos) and microvesicles (MVs) have also shown great potential in regenerative medicine and possessing similar therapeutic functions [41–43]. Stem cells and their derivatives exert therapeutic effects through various mechanisms, including differentiation into target cells, secretion of bioactive factors and modulation of immune responses, with the anti-cellular senescence pathway being particularly crucial [34,44]. In the treatment of OA, stem cells have shown significant effects by slowing down or reversing cellular senescence, reducing the secretion of inflammatory factors, and promoting the regeneration and repair of chondrocytes. Stem cell derivatives, on the other hand, enhance tissue regenerative capacity by delivering beneficial signalling molecules and to some extent, reverses the pathological process and repairs damaged cartilage. Thus, providing new therapeutic avenues and hope for OA treatment.

### 2.3. Anti-ageing effect of mesenchymal stem cells/stromal cells on chondrocytes

#### 2.3.1. Mechanisms of MSCs direct intervention in the regulation of chondrocyte senescence

Studies have confirmed that MSCs can reduce cartilage degradation, pain behaviour, osteophyte formation and joint inflammation [45]. Recent studies have further revealed its deep anti-ageing mechanism:

① Regulation based on Sirtuins (SIRT): SIRT is a NAD + dependent enzyme family that is widely believed to regulate ageing-related signalling pathways, and sirtuin deficiency will accelerate cellular senescence [46,47]. Among them, SIRT1 has been confirmed to regulate cellular senescence and ageing-related processes [48]. Recently, experiments have found that MSCs with high SIRT1 expression can inhibit chondrocyte senescence and SASP production, thereby preventing OA [49] SIRT6 deficiency can aggravate chondrocyte senescence and OA progression, while intra-articular injection of adenovirus-SIRT6 can activate SIRT6, leading to significant relief of OA symptoms and reduction of chondrocyte senescence [50,51]. Taken together, these results suggest that the SIRT family is widely involved in chondrocyte senescence in OA. Therefore, regulating their expression has the potential to improve chondrocyte ageing [50,51].

② Direct repair and mitochondrial transfer: BM-MSCs can transfer healthy mitochondria to OA chondrocytes through direct cell-to-cell contact to replace damaged mitochondria, thereby restoring mitochondrial membrane potential, inhibiting apoptosis and promoting matrix synthesis [52]. Similarly, it has been found that regulating SIRT3 expression can reduce the senescence of OA chondrocytes, accompanied by the improvement of mitochondrial function [53]. Similarly, Lin et al. found that SIRT4 knockdown impaired the ability of chondrocytes to remove damaged mitochondria, increased the accumulation of ROS and promoted chondrocyte senescence. However, upregulation of SIRT4 can enhance mitophagy, restore mitochondrial function and prevent chondrocyte senescence [54]. In addition, SOD secreted by MSCs can also inhibit the P53/P21 senescence signalling pathway by scavenging ROS, and directly alleviate oxidative stress-induced senescence of chondrocytes [55].

③ Cell Rejuvenation: according to the possible ageing phenotype of autologous MSCs (especially in elderly patients), pretreatment with curcumin [56] or rapamycin [57] can activate FoxO3/autophagy pathway or inhibit mTOR pathway, and significantly improve the anti-ageing ability of MSCs themselves. Pretreatment with the senesolytic drug ABT-263 can selectively remove the senescent population of MSCs and reduce the secretion of SASP factors (such as MMP-13 and IL-6), thereby greatly improving their chondrogenic differentiation potential [58].

④ Optimized selection for specific pathological phenotypes: for abnormal angiogenesis in OA pathology, iMSCs are more able to avoid the risk of aggravating tissue inflammation compared with primary BM-MSCs due to their unique phenotype of low pro-angiogenesis [36]. In the case of metabolic OA induced by high-fat diet, studies warn that direct injection of MSCs may activate negative immune responses due to the local chronic inflammatory environment, and the applicability of cellular intervention should be carefully evaluated at this time [45].

#### 2.3.2. Anti-chondrocyte ageing effect of MSCs from different sources

As mentioned above, MSCs can be further classified according to their source, such as BM-MSCs, AD-MSCs and UC-MSCs. Experiments have shown that BM-MSCs, when injected into young mice, exert anti-ageing effects and prolong the lifespan and health of the recipients [59]. Moreover, BM-MSCs fusion materials implanted into the equine cartilage defect model can restore the lubrication function of joint synovial fluid for a long time, reduce cartilage wear and reduce the risk of post-traumatic OA [60]. More importantly, Al-Najar et al. observed a significant improvement in the average thickness of knee cartilage measured by MRI in OA patients 24 months after intra-articular injection of BM-MSCs [61]. Further study confirmed that BM-MSCs can be induced ageing chondrocytes apoptosis, reducing SA - beta - gal positive cells proportion, decrease the sasp related interleukin (IL)-6, IL-1 beta, the expression of MMP-1 and MMP-13, and stimulate the cartilage cell proliferation [16]. In particular, MMPs are responsible for the degradation of extracellular matrix (ECM) proteins in cartilage, including sulphated proteoglycan, collagen and fibronectin, and the loss of cartilage ECM is a key early feature of OA [62]. Studies to 1:1 proportion of BM-MSCs and cartilage cells co-culture, can through paracrine effect and direct interaction between cells, significantly improve OA chondrocytes matrix synthesis ability, inhibit degeneration and fibrosis [63]. This further suggests that BM-MSCs can regulate the pathological development of OA by reducing the multi-target downstream effects of chondrocyte senescence. In addition to inhibiting chondrocyte apoptosis, BM-MSCs can also improve mitochondrial dysfunction by directly promoting chondrocyte proliferation by transferring mitochondria into chondrocytes [52].

The state of BM-MSCs itself also has a significant effect on the therapeutic effect. Normal BM-MSCs can improve the senescence of human/mouse OA-related chondrocytes by inhibiting the accumulation of p16^+^ cells and SASP secretion. However, senescent BM-MSCs not only lose the cartilage protection by highly expressing p16, p21 and SASP factors (DKK1, IL-8, etc.), but also by highly expressing p16, p21 and SASP factors. It can also induce OA in young mice [64]. Even without *in vitro* expansion and scaffold assistance, freshly isolated uncultured BM-MSCs can effectively repair cartilage injury in rabbit ACLT (anterior cruciate ligament transaction) model through a single intra-articular injection, and simplify the clinical treatment process [65]. Intra-articular injection of autologous BM-MSCs in the treatment of patients with stage II-III KOA showed a significant increase in cartilage thickness and continuous improvement in joint function at 24 months of follow-up, with good safety [61].

On the other hand, AD-MSCs have also shown significant anti-ageing and cartilage protection effects. After intra-articular injection of autologous microfragmented adipose tissue (mFAT) rich in AD-MSCs, it can promote the synthesis of cartilage glycosaminoglycan for a long time and relieve pain, and the efficacy is stable for 24 months with good safety [66]. Further studies have shown that AD-MSCs can inhibit cartilage catabolism and inflammatory mediators [67], reduce IL-1β-induced oxidative stress and the expression of senescence markers p21 and p16, thereby slowing down chondrocyte senescence [68]. This was also confirmed by the fact that AD-MSCs co-culture could significantly down-regulate the expression of fibrosis markers induced by inflammation in OA chondrocytes, restore abnormal cell cycle distribution and reduce SA-β-galactosidase positive cases [69]. In premature ageing-like animal models, AD-MSCs delay the ageing process by accelerating mitophagy, eliminating intracellular reactive oxygen species (ROS) and improving mitochondrial quality [59], which are the main pathological changes observed in the progression of OA [70]. In particular, AD-MSCs, administered systemically *via* intravenous administration, can inhibit systemic and joint local inflammation and improve gait abnormalities in guinea pigs with spontaneous OA, which is suitable for multi-joint OA treatment [71].

The therapeutic effect of autologous AD-MSCs is directly related to the stemness of cells. AD-MSCs with high proliferation ability, low ROS level and intact mitochondrial structure can significantly improve the clinical efficacy, and preoperative assessment of cell quality can achieve precise treatment [72]. At the same time, the exploration of specially treated AD-MSCs has also brought new findings. The combination of AD-MSCs and rapamycin can synergistically improve the phenotype of OA chondrocytes. Rapamycin enhances the anti-ageing, anti-inflammatory and cartilage protective functions of AD-MSCs by inhibiting the mTOR pathway, and promotes the secretion of anti-fibrotic factors (FST, THSD4) and pro-survival factor GAS6 [57]. In addition, experiments have confirmed that curcumin pretreatment can rejuvenate oxidative stress-induced AD-MSCs senescence through FoxO3/Beclin1-mediated autophagy, and significantly improve cartilage damage and synovial inflammation in OA model mice [56].

In addition, UC-MSCs have also shown anti-ageing potential. In OA studies, synovial-derived mesenchymal stem cells/stromal cells (SMSCs) with homing peptides can reduce chondrocyte senescence and restore the vitality of damaged cartilage [73]. UC-MSCs are mainly used for OA treatment through cartilage repair and regeneration [74]. The allogeneic transplantation of UC-MSCs into the mouse OA model can delay joint space narrowing by up-regulating anti-inflammatory genes such as indoleamine 2,3-dioxygenase (IDO) and TSG6, without immune rejection [75]. Molecular experiments confirmed that superoxide dismutase (SOD) secreted by UC-MSCs was the core functional molecule in improving chondrocyte ageing, and its anti-ageing effect disappeared after silencing SOD gene [55]. In addition, recently, serum-free UC-MSCs can effectively reduce OA-related cartilage damage and inhibit intra-articular inflammation, with better safety than serum-cultured cells, avoiding the risk of xenogeneic protein contamination and optimizing its application [76].

The anti-ageing effect of other new sources of MSCs has also been verified: the combination of Infrapatellar fat pad derived mesenchymal stem cells/stromal cells (IPFP-MSCs) and platelet-rich plasma (PRP) can effectively repair partial thickness cartilage defect (PTCD) in rats, prevent the defect from developing into irreversible OA and provide a new plan for cartilage protection in the early stage of OA [77]. Wharton glial mesenchymal stem cells/stromal cells (MSC-WJ) can improve the cartilage injury and inflammatory microenvironment of monosodium iodoacetate (MIA) rats by secreting anti-inflammatory factor IL-4 and inhibiting NF-κB signalling pathway [78]. Dpscs-derived conditioned media (CM) can promote the function maintenance of OA chondrocytes by up-regulating the expression of TIMP-1, reducing oxidative stress damage and improving the insufficient synthesis of cartilage matrix [38]. Amniotic membrane mesenchymal stem cells/stromal cells (AMSCs) can improve mandibular osteoporosis by regulating the balance of ageing-related signalling pathways and oxidative stress, which provides a potential treatment idea for OA subchondral bone disease [79].

The exploration of various sources of MSCs to improve the efficacy and mechanism of anti-cellular senescence in OA has provided us with broader ideas and great clinical application potential (Table 1).

**Table 1.** Direct use of mesenchymal stem cells for the treatment of osteoarthritis through anti-aging therapy has been reported in research.

Source of mesenchymal stem cells	Target cells/tissues	Intervention effects	References
AD-MSCs	Human OA Chondrocytes, Peripheral Blood Mononuclear Cells (PBMCs)	Inhibit the mTOR pathway and downregulate senescence markers (p15^INK4B); reduce the senescent phenotype of OA chondrocytes and improve the cartilage microenvironment	[57]
Autologous AD-MSCs	Knee Joints of OA Patients	AD-MSCs with high stemness exhibit low senescence levels (low proportion of β-galactosidase-positive cells and low ROS levels) and better cartilage repair effect; screening low-senescence AD-MSCs can improve treatment success rate	[72]
BM-MSCs	Human OA Chondrocytes, Mouse OA Joints	Normal BM-MSCs inhibit the accumulation of p16⁺ senescent cells and the secretion of senescence-associated secretory phenotype (SASP); downregulate senescence markers (p16, p21) and catabolic genes of OA chondrocytes	[64]
AD-MSCs	Mouse AD-MSCs, Mouse OA Model Joints	Activate the FoxO3/autophagy pathway and downregulate senescence markers (Cdkn1a, Cdkn2a); reduce ROS production, improve oxidative stress-induced senescence of AD-MSCs, and indirectly protect joint tissues	[56]
UCB-MSCs	Human OA Chondrocytes, Rabbit OA Joints	Secrete SOD to scavenge ROS and inhibit the P53/P21 pathway; reduce the SA-β-galactosidase-positive rate of OA chondrocytes and downregulate senescence markers (P21, P53)	[55]
DPSCs, BM-MSCs	Mouse Immature Articular Chondrocytes (iMACs)	DPSCs conditioned medium improves the pathological senescence-related phenotype of iMACs; reduces oxidative stress damage, enhances cartilage matrix synthesis, and alleviates cellular dysfunction	[38]
ARDE (containing AD-MSCs)	Temporomandibular Joint OA (TMJOA) Joints, Rabbit Mandibular Condylar Chondrocytes	Significantly reduce the proportion of SA-β-galactosidase-positive cells and alleviate IL-1β/TNF-α-induced chondrocyte senescence; restore abnormal cell cycle distribution and inhibit senescence-associated fibrosis	[69]
BM-MSCs	Rat Senescent Chondrocytes, OA Joint Cartilage Defects	ABT-263 clears senescent chondrocytes in the joint and improves the senescent microenvironment; reduces apoptosis and senescence of BM-MSCs (decreased SA-β-Gal positive rate) and enhances cartilage regeneration capacity	[16]
Synovial MSCs	Autologous Synovial MSCs	Selectively clear senescent cells in synovial MSCs of OA patients and reduce the SA-β-Gal positive rate; decrease the secretion of SASP factors (IL-6, MMP-13) and enhance stem cell stemness	[58]
MSCs	Sheep Meniscal Cartilage Tissue, PBMCs	Long-term *in vitro* passage simulates stem cell senescence; early-passage MSCs have strong chondrogenic differentiation ability, while late-passage MSCs still retain trophic repair function; chondrogenic potential gradually declines during senescence	[172]
MSCs from Various Sources	Human/Rabbit OA Chondrocytes, OA Joints	Improve inflammation-induced senescence of chondrocytes, reverse the senescent phenotype of decreased proliferation and reduced matrix synthesis; inhibit oxidative stress-mediated senescence and apoptosis of chondrocytes	[39]
AMSCs	Mouse Mandible	Downregulate senescence-related molecules (p16, p21, p53) and inhibit oxidative stress; improve cellular senescence in Bmi-1⁻/⁻ mice, promote osteogenesis and inhibit osteoclastogenesis	[79]

AD-MSCs, adipose-derived mesenchymal stem cells; OA, osteoarthritis; PBMCs, peripheral blood mononuclear cells; ROS, reactive oxygen species; sod, superoxide dismutase; BM-MSCs, bone marrow-derived mesenchymal stem cells; SASP, senescence-associated secretory phenotype; AD-MSCs, adipose-derived stem cells; hUCB-MSCs, human umbilical cord blood mesenchymal stem cells; DPSCs, dental pulp stem cells; iMACs, immature articular chondrocytes; ARDE, AD-MSCs-enriched adipose extract; TMJOA, temporomandibular joint osteoarthritis; IL-1β, interleukin-1β; TNF-α, tumour necrosis factor-α; BM-MSCs, bone marrow-derived mesenchymal stem cells; MMP-13, matrix metalloproteinase-13; Synovial MSCs, synovial mesenchymal stem cells; MSCs, mesenchymal stem cells; AMSCs, amniotic membrane mesenchymal stem cells.

#### 2.3.3. Anti-chondrocyte senescence effect of specially modified stem cells

These findings suggest that the anti-chondrocyte senescence effect of MSCs is an important cornerstone for the treatment of OA. However, the poor microenvironment of OA joint composed of complex inflammatory factors, oxidative stress and SASP often limits the survival and function of single-source MSCs. Therefore, empowering MSCs through ‘special modification’ has become the core approach to enhance their anti-ageing efficacy (Table 2).

**Table 2.** Research report on the treatment of osteoarthritis through anti-aging therapy using special processed mesenchymal stem cells.

Stem cell modification methods	Target cells/tissues	Intervention effects	References
Gene Modification - Sirt1-Overexpressing MSCs	Mouse Articular Chondrocytes	Inhibit chondrocyte senescence and SASP secretion, downregulate senescence markers; reduce cartilage matrix degradation and delay joint degeneration mediated by OA-related cellular senescence	[49]
Gene Modification - ALKBH5-Overexpressing BM-MSCs	Human BM-MSCs, Mouse OA Joints	Improve replicative and pathological senescence of BM-MSCs, downregulate senescence markers (p16, p21); reduce mitochondrial dysfunction and ROS accumulation, and alleviate joint tissue senescence	[82]
Gene Modification - DGCR8-Overexpressing MSCs	Human MSCs, Mouse OA Joints	Stabilize heterochromatin and inhibit replicative and pathological senescence of MSCs; reduce the accumulation of p16-positive senescent cells in the joint and delay OA progression	[83]
Gene Modification - YAP/FOXD1-Overexpressing MSCs	Human MSCs, Mouse OA Joints	Inhibit replicative senescence and pathological senescence (e.g. Werner syndrome-related senescence) of MSCs; downregulate the expression of senescence markers (p16, p21) and reduce ROS production	[84]
Hydrogel - SKP@miR Self-Assembling Peptide Hydrogel	Rat Chondrocytes, SMSCs	Inhibit OA-related chondrocyte senescence and downregulate senescence markers (P16^INK4a, P21); improve cellular proliferation capacity and reduce senescence-mediated matrix degradation	[73]
Cytokine Stimulation - hPL-Pretreated UC-MSCs	Human OA Chondrocytes	Secrete IGF2 to activate the autophagy pathway and specifically improve the senescent chondrocyte phenotype; reduce SA-β-Gal activity and p16/p21 expression, and enhance the proliferation capacity of senescent chondrocytes	[88]
Cytokine Stimulation - Stepwise Preconditioned MSCs	Rabbit OA Joints, Human MSCs	Delay replicative senescence of MSCs and reduce the proportion of SA-β-galactosidase-positive cells in late passages; enhance the anti-senescence capacity and cartilage repair potential of stem cells	[89]
Hypoxic Culture - Hypoxia-Cultured UC-MSCs	Rat OA Joints, Chondrocytes	Downregulate senescence markers (p16, p21) and maintain telomerase activity; delay replicative senescence of UC-MSCs and enhance immunomodulatory and cartilage-protective effects	[91]
3D Suspension Treatment - 3D Hanging Drop-Cultured UC-MSCs	Rabbit OA Cartilage Defects, UC-MSCs	Downregulate replication senescence-related genes (TP53, Serpine1); improve *in vitro* culture senescence of UC-MSCs and maintain stem cell stemness and immunomodulatory function	[92]

MSCs, mesenchymal stem cells; BM-MSCs, bone marrow-derived mesenchymal stem cells; OA, osteoarthritis; hPL, human platelet lysate; UC-MSCs, umbilical cord mesenchymal stem cells; IGF2, insulin-like growth factor 2; UC-MSCs, umbilical cord mesenchymal stem cells; SMSCs, synovial mesenchymal stem cells.

① Genetic modification: Genetic modification can directly improve the anti-ageing activity or cartilage protection function of MSCs by precisely regulating key signalling pathways. MSCs overexpressing Sirt1 can significantly rescue the OA phenotype of 1,25 (OH)_2_D deficient mice and inhibit chondrogenic senescence and SASP secretion through the ‘1,25 (OH)_2_D_3_-VDR-Sirt1’ pathway [49]. CPSCs with mitochondrial fusion protein 2 (Mfn2) overexpression can promote chondrogenic differentiation, reduce chondrocyte apoptosis and matrix degradation, and improve OA cartilage damage by inhibiting the Notch2 pathway [80]. Inhibition of miR-21 expression in MSCs (MSC-miR-21^-^) can significantly reduce systemic inflammation and SASP levels in OA model by regulating the ERK1/2/AKT pathway, and the effect is better than cell therapy [81]. BM-MSCs overexpressing m6A demethylase ALKBH5 can reduce DNA damage of chondrocytes, improve mitochondrial function, reduce cartilage degeneration in ACLT mice through IGF2BP1-CYP1B1 regulatory axis [82], and optimize the therapeutic effect of OA. Overexpression of DGCR8 can inhibit premature senescence of MSCs by stabilizing heterochromatin structure, and its N-terminal domain interacts with KAP1 and Lamin B1 to maintain genomic stability and delay the progression of OA [83]. The activation of YAP-TEAD-FOXD1 signalling axis can inhibit the replicative senescence and pathological senescence of MSCs, reduce the accumulation of senescent cells in the joint and promote cartilage repair [84]. After silencing the SOD gene of MSCs, its anti-ageing effect of scavenging ROS and inhibiting P53/P21 pathway completely disappeared, confirming that SOD is the core secreted factor of MSCs in improving chondrogenic ageing [55].

② Hydrogel composite modification: hydrogel as a carrier can improve the retention rate of MSCs *in vivo*, protect the cell activity and play a synergistic anti-ageing effect. Amniotic hydrogel combined with AD-MSCs plays a synergistic anti-inflammatory and anti-degradation effect by inhibiting Wnt/β-catenin pathway to reduce the loss of cartilage matrix, and the effect is better than that of single intervention [67]. The combination of arthroscopic irrigation-derived MSCs and hyper-branched poly(ethylene glycol) diacrylate (HB-PEGDA)/HA hydrogel can rapidly gel to fill cartilage defects. The three-dimensional microenvironment of the hydrogel promotes the chondrogenic differentiation of MSCs and significantly improves the cartilage repair effect [85]. Functionalized self-assembling peptide nanofiber hydrogel loaded with miR-29b-5p can achieve sustained release (40 days *in vitro*), and at the same time, it can attract endogenous SMSCs and significantly reduce the expression of chondrocyte senescence markers (P16^INK4a, P21) [73]. GelMA hydrogel loaded with targeted lipid nanoparticles can prolong the action time of siCH25H in the joint, improve cholesterol metabolism disorder of chondrocytes and indirectly alleviate the decline of cell function [86]. Allogeneic UC-MSCs combined with hyaluronic acid (HA), combined with HTO surgery to correct the force line, can promote the repair of large area of cartilage defects and the effect of cartilage repair is stable during long-term follow-up [74].

③ Cytokine stimulation/pretreatment: MSCs can be ‘rejuvenated’ and their anti-ageing and repair potential can be enhanced by pretreatment with cytokines or chemical reagents. Ngf-sf/CS-BM-MSCs complex promoted subchondral bone repair through nerve growth factor (NGF) sustained-release microspheres, indirectly improved the microenvironment of cartilage regeneration and provided mechanical support and nutritional supply for chondrocytes [87]. UC-MSCs pretreated with human platelet lysate (hPL) can secrete insulin-like growth factor 2 (IGF2) by increasing glutathione (GSH) level and activate autophagy pathway to specifically rejuvenate the ageing phenotype of OA chondrocytes [88]. Stepwise pretreatment (7 days of chondrogenic medium combined with normal medium) can improve the proliferation and chondrogenic differentiation potential of MSCs derived from elderly patients and a single high dose injection can significantly alleviate OA cartilage damage [89]. Pretreatment of CPSCs with a mitochondrial fusion promoter (MFP1) can induce mitochondrial fusion, up-regulate the expression of chondrogenic differentiation markers, enhance matrix synthesis and have a synergistic anti-ageing effect with the overexpression of Mfn2 [90].

④ Hypoxia: long-term hypoxia (1% O_2_) can significantly optimize the anti-ageing properties of MSCs. Hypoxia-preconditioned (Hypo)-induced UC-MSCs maintained stemness through hypoxia-inducible factor (HIF)-1α, maintained high proliferation ability and low senescence level (down-regulated expression of p16 and p21) for 30 generations, and had stronger immunoregulatory ability, which significantly inhibited the activity of CD3^+^CD8^+^T cells and promoted the proliferation of regulatory T cell (Treg). At the same time, it can protect chondrocytes from inflammation-induced apoptosis and alleviate OA joint swelling and cartilage damage [91].

⑤3D culture: 3D culture can simulate the *in vivo* microenvironment and improve the anti-ageing and repair function of MSCs. 3D hanging drop cultured UC-MSCs can down-regulate ageing related genes such as TP53, up-regulate immunomodulatory genes (TSG6, IDO1) and homing genes (CXCR4), and its cartilage repair effect is significantly better than that of 2D cultured cells [92]. Human embryonic stem cell-derived mesenchymal stem cell spheroid (EMSCₛₚ) remained highly active after 7 days of delivery at room temperature and was more tolerant to joint cavity hypoxia and low nutritional environment than single cell. By enhancing HIF family genes, the EMSCₛₚ showed better cartilage repair effect in the rhesus macaque model of spontaneous OA [37].

⑥ Scaffold-assisted modification: Scaffold materials can provide colonization support for MSCs and synergistically improve the anti-ageing treatment effect of OA. The NGF into a silk fibroin/chitosan (NGF-SF/CS) porous scaffold loaded BM-MSCs and NGF sustained-release microspheres. The honeycomb-like structure of the scaffold promoted cell adhesion and proliferation, and the sustained release of NGF promoted subchondral bone repair and indirectly improved cartilage regeneration [87]. In the *in vivo* experiment, BM-MSCs were embedded in the low-gel temperature agarose gel scaffold for transplantation, which was helpful for the colonization of stem cells in the cartilage defect site [16]. MSCs and platelet-rich fibrin composite scaffolds were implanted into equine cartilage defects. Fibrin provided MSCs with a scaffold for colonization, which could restore the lubrication function of joint synovial fluid for a long time, reduce the mechanical wear of cartilage, and reduce the risk of post-traumatic OA [60]. 3D bioprinted GelMA-MSCs scaffold combined with miR-410 can accurately match the morphology of cartilage defects. miR-410 enhances MSCs migration and chondrogenic differentiation by inhibiting Wnt3a pathway to achieve cartilage-bone integrated repair [93]. 3D water-phase silk fibroin scaffold combined with MSCs can form cartilaginous tissue under the action of cartilage inducers. Its porous structure promotes intercellular interaction, up-regulates cartilage markers such as Col-II and Aggrecan, and inhibits osteogenic differentiation and fibrosis [94]. The collagen cell carrier (CCC) scaffold loaded with chondrocytes differentiated from AMSCs can maintain hyalin phenotype, with high expression of collagen II and lubricin and low expression of apoptosis markers, providing functionally complete repair materials for cartilage defects in OA [95].

⑦ Other combined applications: Needle knife combined with human AD-MSCs can promote the proliferation of chondrocytes, reduce cartilage matrix degradation, and release the adhesion around the knee to improve the joint mechanical environment by regulating GSK3β-cyclin D1-CDK4/CDK6 signalling pathway, so as to synergistically relieve the symptoms of OA [96]. The combined application of MSCs and high tibial osteotomy (HTO) [66,74,97] can significantly improve the clinical scores (WOMAC, VAS) and increase the cartilage thickness detected by MRI.

### 2.4. The anti-chondrocyte senescence effect of mesenchymal stem cell derivatives in OA

In addition to direct use of stem cells, their derivatives are becoming an emerging therapeutic method (Table 3). These derivatives not only have significant advantages in anti-ageing, but also effectively overcome the limitations of immune rejection, tumourigenesis risk, storage and transportation inconvenience caused by stem cell transplantation due to their cell-free therapy [35]. Studies have shown that mesenchymal stem cell derivatives have shown great potential in the treatment of OA by delivering bioactive molecules, inhibiting inflammatory response, reducing oxidative stress and regulating cell metabolic balance.

**Table 3.** Research on the treatment of osteoarthritis through mesenchymal stem cell derivatives by anti-cell aging methods.

Source of mesenchymal stem cells	Target cells/tissues	Intervention effects	References
Adipose-Derived AD-MSCs (CM, MV, Exos)	Human OA Osteoblasts	Reduce IL-1β-induced SA-β-Gal activity and γH2AX foci (DNA damage); alleviate oxidative stress-mediated senescence and restore mitochondrial membrane potential; downregulate pro-inflammatory factors and upregulate IL-10	[100]
Umbilical Cord Matrix MSCs-EVs	Rat OA Joints, Autologous Synovial MSCs	Inhibit the secretion of SASP factors (e.g. IL-6, MMP-9); improve systemic inflammation, reduce senescent cell-related damage in joints, and indirectly regulate cellular senescence	[81]
Equine AD-MSCs-EVs	Equine AD-MSCs, OA-Related Chondrocytes	Enrich anti-senescence miRNAs (e.g. eca-miR-29b); presumably inhibit chondrocyte senescence and regulate the NF-κB pathway as well as MMPs expression	[110]
EMSCs-EVs	Human OA Chondrocytes, Mouse OA Joints (Post-Traumatic/Naturally Aging Models)	Activate the FOXO1A-autophagy axis; decrease P1614ᵏ4ᵃ expression, SA-β-Gal positive rate, and γH2AX (DNA damage); rejuvenate senescent chondrocytes and restore their proliferation and matrix synthesis functions	[107]
Young Mouse Bone Marrow-Derived BM-MSCs-YEVs	Human Primary OA Chondrocytes, Mouse OA Joints (DMM Model)	Reduce the SA-β-Gal positive rate and the expression of p16 and p21 in chondrocytes; activate the PTEN/PI3K/AKT pathway and CLEAR signaling pathway, improve mitochondrial function, and inhibit SASP release	[106]
AD-MSCs-EVs	Human OA Chondrocytes (DNA Damage/Inflammation-Induced Senescence Models)	Dose-dependently reduce the SA-β-Gal positive rate and γH2AX foci; downregulate p15, p21, and p27, inhibit the secretion of SASP factors (e.g. IL6, MMP-13); upregulate cartilage synthesis markers	[111]
Bone Marrow-Derived BM-MSCs (Hypoxia-Pretreated HCM/HEVs)	Porcine Chondrocytes, Human BM-MSCs, Macrophages	Inhibit IL-1β-induced chondrocyte senescence (decreased SA-β-Gal staining positive rate); promote matrix synthesis, inhibit catabolism, and improve the senescent microenvironment	[104]
Bone Marrow-Derived MSCs-CM	Human ONFH Bone Marrow Cells, Bone Tissue of Mouse Ischemic Osteonecrosis Model	Inhibit the expression of senescence markers such as p16^INK4a, p21, and p53; reduce SASP factors (e.g. IL-6, MMP-3), indirectly providing a basis for the regulation of OA cellular senescence	[144]
Bone Marrow-Derived MSCs-CM (P5 Young Stem Cells)	Human MSCs, Bone Tissue of Mouse Postmenopausal Osteoporosis Model	Decrease SA-β-Gal activity and SASP factor expression; slightly extend telomere length, improve stem cell senescent phenotype, and indirectly correlate with OA cellular senescence	[147]
iMSCs-CM	Human OA Chondrocytes	Reduce the SA-β-Gal positive rate and p21 protein expression; decrease ROS production and DNA double-strand breaks, delay chondrocyte senescence, and maintain the cartilage phenotype	[145]
Umbilical Cord UC-MSCs-dECM	Rat Chondrocytes (H₂O₂-Induced Senescence), Rat OA Joints (ACLT Model)	Inhibit the STING-NF-κB pathway; reduce the expression of senescence markers P16 and P21, and alleviate degeneration mediated by chondrocyte senescence	[142]
Human Synovial Fluid SMSCs-MEV (Mitochondria-Enriched)	Human Stress Chondrocytes (IL-1β-Induced), Rat OA Joints (DMM Model)	Downregulate the expression of p16, p21, p53, and CAV1; upregulate the anti-senescence protein SIRT1; restore mitochondrial function, reduce ROS accumulation, and inhibit cellular senescence	[173]
Human iMSCs-MSC-sEVs (WPD-Modified + siMDM2-Loaded)	Human OA Chondrocytes, Mouse OA Joints (ACLT/Naturally Aging Models)	Target and clear senescent chondrocytes, reducing the SA-β-Gal positive rate from 73.78% to 31.13%; downregulate P16^INK4a and SASP factors (e.g. IL-6, TNF-α), and activate the MDM2-P53 pathway	[113]
Mouse Bone Marrow BM-MSCs-FTO-EVs (FTO-Overexpressing)	Mouse Chondrocytes (LPS-Induced Inflammatory Senescence), OA Joints (MIA Model)	Reduce the SA-β-Gal positive rate and the expression of p16 and p21; activate the Nrf2-HO-1 antioxidant pathway and inhibit oxidative stress-mediated senescence	[112]
Human Bone Marrow BM-MSCs-ML-EVs (Mechanical Load + miR-27b-3p Overexpression + CTP Modification)	Mouse Senescent Chondrocytes (TBHP-Induced), OA Joints (DMM Model)	Inhibit the ROR1/NF-κB pathway; reduce the expression of p16 and p21 as well as SA-β-Gal activity; restore the proliferation and matrix synthesis functions of chondrocytes	[114]
Healthy Human AD-MSCs-H-EVs	Human OA Chondrocytes, Synovial Cells, Mouse OA Joints (CIOA Model)	Reduce the proportion of SA-β-Gal positive cells and the expression of p15; induce M2 polarization of macrophages, inhibit SASP factor secretion, and improve the joint senescent microenvironment	[108]
Human AD-MSCs-EVs (IL-1β-Pretreated)	Human OA Cartilage Explants	Contain anti-senescence miRNAs (e.g. miR-449b-5p); target SIRT1 and PRKAA1, regulate the chondrocyte senescence process, and inhibit apoptosis	[115]
Mouse MSC-MVs (IFN-γ-Pretreated) + ROS-Responsive Hydrogel	Mouse Chondrocytes (H₂O₂-Induced Senescence), Rat OA Joints (DMM+ACLT Model)	Reduce the SA-β-Gal positive rate and the expression of p16, p21, and p53; improve the balance of mitochondrial fission/fusion, reduce ROS accumulation, and inhibit cellular senescence	[54,59]
Mouse MSC-iExos (Hsp70-Enriched) + Liposomes	Mouse Chondrocytes (H₂O₂-Induced Senescence), Rat OA Joints (DMM+ACLT Model)	Downregulate p16, p21, and p53; restore mitochondrial membrane potential, maintain mitochondrial morphology, and exert anti-senescence effects through the Hsp70-AMPK-SIRT3 axis	[131]
Rat Bone Marrow BM-MSCs-Hypo-Exos (Hypoxia-Pretreated)	Rat OA Chondrocytes (IL-1β-Induced), OA Joints (ACLT+MMx Model)	Reduce the expression of p16, p21, p53, and the SA-β-Gal positive rate; decrease ROS production and delay inflammation-induced senescence of chondrocytes	[132]
Rat Bone Marrow BM-MSCs-MVs/Mitochondria	Rat OA Chondrocytes (IL-1β-Induced), OA Joints (Collagenase-Induced Model)	Activate the PGC-1α signaling pathway and promote mitochondrial biogenesis; reduce chondrocyte apoptosis and senescence-related dysfunction, and improve mitochondrial dysfunction	[135]
Rat Adipose AD-MSCs-MVs + Chitosan/Gelatin Hydrogel	Rat OA Chondrocytes (IL-1β-Induced), Mouse OA Joints (DMM Model)	Activate the SIRT1/FOXO3a pathway; reduce the expression of p16 and p21 as well as the SA-β-Gal positive rate, decrease ROS accumulation, and delay cellular senescence	[137]
Human Adipose AD-MSC-MV/Exos	Human OA Chondrocytes (IL-1β-Induced)	Inhibit inflammation-mediated cellular senescence and reduce the number of SA-β-Gal positive cells; downregulate MMP-13, upregulate Collagen II, and improve the function of senescent chondrocytes	[174]

OA, osteoarthritis; MSCs, mesenchymal stem cells; AD-MSCs, adipose-derived stem cells; CM, conditioned medium; MV, microvesicles; Exos, exosomes; EVs, extracellular vesicles; EMSCs-EVs, embryonic stem cell-derived small extracellular vesicles; BM-MSCs-YEVs, bone marrow-derived mesenchymal stem cells-derived young extracellular vesicles; DMM, destabilization of the medial meniscus; HCM, hypoxia-preconditioned conditioned medium; HEVs, hypoxia-preconditioned extracellular vesicles; ONFH, osteonecrosis of the femoral head; iMSCs, induced pluripotent stem cell-derived mesenchymal stem cells; UC-MSCs-dECM, umbilical cord mesenchymal stem cells-derived decellularized matrix; ACLT, anterior cruciate ligament transaction; SMSCs-MEV, synovial fluid-derived mesenchymal stem cells-derived mitochondria-enriched extracellular vesicles; CAV1, caveolin-1; SIRT1, sirtuin 1; WPD, a specific engineering modification; FTO-EVs, fat mass and obesity-associated protein-overexpressing extracellular vesicles; LPS, lipopolysaccharide; MIA, monosodium iodoacetate; ML-EVs, mechanical load-pretreated extracellular vesicles; CTP, chondrocyte-targeting peptide; TBHP, tert-butyl hydroperoxide; CIOA, collagen-induced osteoarthritis; IFN-γ, interferon-γ; iExos, interferon-γ-pretreated exosomes; Hypo-Exos, hypoxia-pretreated exosomes; PGC-1α, peroxisome proliferator-activated receptor gamma coactivator 1 alpha; AD-MSCs-MVs, adipose-derived stem cells-derived microvesicles; MMP, matrix metalloproteinase; IL, interleukin; SASP, senescence-associated secretory phenotype; TNF-α, tumour necrosis factor-α; ROS, reactive oxygen species; STING, stimulator of interferon genes.

#### 2.4.1. Extracellular vesicles (EVs)

EVs are one of the most widely studied types of stem cell derivatives. They precisely regulate OA-related cellular senescence by delivering miRNA, proteins and other active components, and their function is significantly affected by the source of donor and pretreatment methods. EVs can be classified into various subtypes in research: Exos (30–150 nm in diameter), MVs (150–1000 nm in diameter) and apoptotic bodies (>1000 nm in diameter) [98], but many reports have explored evs as a whole, and the following will discuss the intervention effects and mechanisms of evs as a whole [98].

Similar to MSCs, EVs from different sources have certain differences in their efficacy: EVs derived from AD-MSCs promote the proliferation and migration of human OA chondrocytes, maintain cartilage matrix and down-regulate IL-1β-induced senescent-related SA-β-gal activity and γ-h2ax lesion accumulation [99,100]. AD-MSCs-EVs can also be efficiently taken up by synovial fibroblasts (FLSs) through the CD44-HA axis, deliver functional mirnas such as hsa-miR-191, and target the inhibition of the NF-κB pathway. It can significantly down-regulate the expression of pro-inflammatory factors such as IL-6 and chemokine (C-C motif) ligand 2 (CCL2) and matrix degrading enzymes such as MMP-1 and MMP-3 [101]. msc-ev [102] from patellar fat pad can reduce pain, inhibit cartilage degeneration, osteochondroma formation, and synovial inflammation, thereby delaying the progression of OA [102]. After purification by anion exchange chromatography, MSC-EVs can improve gait abnormality, reduce cartilage erosion, and inhibit M1 macrophage infiltration in mouse ACLT model. The effect of MSC-EVs is better than that of simple scaffold treatment [102]. In the coccygeal intervertebral disc degeneration (IDD) rat model, BM-MSC-EVs enhanced the proliferation of degenerative intervertebral disc cells, reduced senescence and alleviated intervertebral disc degeneration [103], reflecting its potential therapeutic value in cartilage repair. Similar molecular studies have shown that BM-MSC-EVs can regulate the function of chondrocytes, prevent cartilage destruction, inhibit abnormal subchondral bone metabolism and synovial tissue changes, thereby improving OA joint pain [45,104]While promoting chondrocyte proliferation, migration and matrix deposition, BM-MSC-evs can also inhibit IL-1β-induced chondrocyte senescence, apoptosis and matrix degradation [104,105]. Young mesenchymal stem cells/stromal cells -derived extracellular vesicles (YEVs) derived from young BM-MSCs significantly reduced the positive rate of SA-β-gal and the expression of p16 and p21 in OA chondrocytes by activating PTEN signalling pathway and inhibiting PI3K/AKT pro-ageing pathway, while EVs derived from old BM-MSCs had no obvious anti-ageing effect. Clarifying the donor age is a key determinant of the efficacy of EVs [106]. EMSCs -sEVs rejuvenate non-early OA chondrocytes and reduce P16^INK4a positive cells and SASP factor release by activating FOXO1a-autophagy axis, which is effective in both post-traumatic OA and natural ageing OA models [107]. In addition, studies have also shown that hUC-MSC-EVs can reduce SA-β-Gal activity and γ-h2ax foci in chondrocytes, and significantly reduce the levels of IL-6 and tumour necrosis factor (TNF)-α [81]. Although MSC-evs from various sources have good anti-ageing effects, studies have shown that the existing senescent MSC-evs are defective and ineffective in the prevention and [108] of OA [108].

The anti-ageing effect of EVs is mainly achieved through multiple pathways: regulating inflammatory signalling pathways (such as NF-κB and MAPK) and reducing the secretion of pro-inflammatory factors [109]; Delivery of anti-ageing mirnas (such as miR-29b and miR-146a) to inhibit the expression of chondrocyte ageing markers [110]; Repair mitochondrial function, reduce ROS accumulation and DNA damage [111]; Regulation of autophagy pathway, removal of damaged organelles, and maintenance of cell homeostasis [107]. 此 In addition, BM-MSCs-derived EVs target TLR1/4 inflammatory pathway through miR-126-3p and inhibit chondrocyte senescence [104].

As a cell-free therapy, EVs have low immunogenicity and high safety, and can accurately target the lesion site through intra-articular injection [99]. EVs from fat, infrapatellar fat pad and other sources are easy to prepare and can be obtained from surgical waste, which is suitable for large-scale production [101,102].

Functional enhancement of EVs by special modification and pretreatment has become the core direction to improve the therapeutic efficacy of OA. Different modification strategies significantly optimize the therapeutic effect by precisely regulating the anti-ageing pathway.

① Through genetic modification, the loading of functional nucleic acid molecules can be regulated to accurately regulate the ageing signalling pathway. Fto-enriched EVs (FTO-EVs) activate autophagy-related gene (ATG) 5/ATG7-mediated autophagy through m6A demethylation modification, inhibit lipopolysaccharide (LPS)-induced chondrocyte senescence, and reduce the expression of p16 and p21 [112]. The engineered vesicles WPD-sEVs^siMDM2 were loaded with siMDM2 by electroporation, silenced the MDM2 gene to activate the P53 pathway, and specifically eliminated senescent chondrocytes, reducing the proportion of senescent cells from 73.78% to 31.13% [113]. Mechanical loading-primed (ML)-EVs pretreated with mechanical loading can target the ROR1/NF-κB pathway, reduce the expression of p16/p21, and restore the proliferation function of chondrocytes due to the enrichment of miR-27b-3p [114]. The core potential of this modification strategy is to achieve the dual effect of ‘precise targeting + signalling pathway regulation’ and avoid off-target side effects.

② Optimize the functional components of EVs by pretreating donor cells with cytokines. IL-1β pretreated AD-MSCs-EVs enriched anti-inflammatory and anti-ageing mirnas such as miR-146a-5p, targeted regulation of Wnt and transforming growth factor-beta (TGF-β) pathways, and inhibited abnormal ageing of chondrocytes [115]. HEVs derived from healthy AD-MSCs are rich in anti-inflammatory proteins such as apolipoprotein E, which can induce the polarization of M2 macrophages and reduce oxidative stress and inflammatory senescence of synovial cells. However, S-EVs derived from senescent AD-MSCs aggravate the progression of OA due to defective functional components [108]. MVs pre-stimulated by interferon (IFN)-γ loaded on ROS-responsive hydrogel can improve the ageing phenotype of chondrocytes by regulating the balance of mitochondrial division/fusion in response to the intelligent release of local high ROS microenvironment in OA [54,59]. This strategy activates the donor cells by simulating the inflammatory microenvironment, making EVs more suitable for the pathological state of OA and more targeted for treatment.

③ Physical preconditioning such as mechanical loading and hypoxia can optimize the functional components of EVs. After human BM-MSCs were treated with cyclic mechanical loading, miR-27b-3p was enriched in EVs, which could simulate the anti-ageing effect of cytokine pretreatment, and the effect disappeared after silencing miR-27b-3p [114]. Hypoxia-pretreated BM-MSCs-EVs further enhance the anti-chondrocyte ageing effect by enhancing the inhibition of TLR1/4 inflammatory pathway [104]. The physical pretreatment method is simple and easy to perform, without complex gene editing, and is easier to achieve clinical large-scale production.

#### 2.4.2. Exosomes

Exos are small molecular EVs with a diameter of 30–150nm. Due to their strong stability and enrichment of active components, they have shown unique advantages in the anti-ageing treatment of OA, and there are significant differences in the efficacy and mechanism of Exos from different sources [116,117].

Hypoxia-induced AD-MSCs-Exos can inhibit IL-1β-induced [118] chondrocyte senescence and SASP expression, and reduce the degradation of OA articular cartilage [118]. If AD-MSCs-Exos highly express miR-376c-3p, Mir-376c-3p can further inhibit WNT-β-catenin pathway by targeting WNT3 and WNT9a, reduce MMP-13 and ADAMTS5 expression, and inhibit inflammation and fibrosis of FLSs [119]. Exos derived from subcutaneous AD-MSCs enhance autophagy of ageing OA chondrocytes and promote the repair of damaged cartilage [119]. iMSCs-MSC-Exos can inhibit the TNF-α/NF-κB pathway [105], and promote the proliferation and migration of chondrocytes [120]. The anti-inflammatory activity of iMSC-Exos derived from iMSCs is not affected by long-term culture. Compared with BM-MSCs-Exos, imSC-Exos is more suitable for large-scale production, and highly expresses surface markers such as CD29 and CD44 to enhance the interaction with chondrocytes [120]. Deer horn AD-MSCs-Exos can reverse stem cell ageing phenotype by delivering proliferation related proteins, activating cell division and DNA repair pathways, and at the same time alleviate cartilage degeneration and bone damage in mouse ACLT model [121]. BM-MSCs-exos can maintain the chondrocyte phenotype by inhibiting chondrocyte senescence and apoptosis, reducing cartilage destruction, alleviating joint damage, and restoring bone trabeculae in OA [122] rats [122]. Some studies compared BM-MSCs-Exo with AD-MSCs-Exo, and found that BM-MSCs-Exo had better effects in promoting the expression of COL2A1 and Sox9 in mouse OA model, which provided a basis for the selection of clinical Exos sources [123]. UC-MSCs-Exos can also inhibit the production of SASP by inhibiting the production of ROS and apoptosis of OA chondrocytes [124,125].

Exos target chondrocyte ageing and matrix metabolic balance through the precise delivery of miRNA, protein and other active ingredients [126]. Compared with EVs, Exos have more uniform particle size and stronger stability, and can be engineered to enhance the anti-ageing effect (such as electroporation loading miRNA) [105]. CPC-Exos promote the proliferation and migration of chondrocytes and maintain the homeostasis of cartilage matrix by transporting miR-221-3p, among which MRL-EVs derived from ‘super healing’ mice have a better effect [126]. BM-MSCs-Exos can indirectly improve the function of chondrocytes and reduce inflammation-mediated ageing by regulating the M1→M2 polarization of synovial macrophages [127].

Recently, a number of studies have attempted to further improve the effect of anti-ageing therapy through special treatment of multi-dimensional Exos:

① Precise regulation of ageing-related pathways by loading miRNA or siRNA. By electroporating miR-140-5p, hiSC-MSCs-EXos target inhibit the TNF-α/NF-κB pathway and significantly enhance the expression of COL2A1 and ACAN in OA chondrocytes, and the effect is better than that of unmodified Exos [105]. Hucmsc-exos can target the 3′utr region of NOX4 by transfection of miR-100-5p mimics, inhibit NOX4 expression, reduce ROS generation, and improve mechanical load-induced chondrocyte damage [125]. Genetic modification made outside secrete body ‘precise targeting carrier’, to provide individualized treatment of OA.

②through the coupling surface target molecules or carrier recombination, promote efficiency of targeted delivery. Cap-cd56 ^+^CD271^+^ BM-MSCs Exos were modified with chondrocyte-specific antigen peptide (CAP) and loaded on PVA/SA composite hydrogel to prolong the retention time of Exos at the injured site and inhibit HIF-3α through miR-210-3p. The expression of p16 and p21 was down-regulated [128]. CAP-EXO coupled with CAP-targeting peptide, encapsulated in HA-SH microgel, increased the binding ability of chondrocytes by more than 3 times, prolonged the local retention time of joints to more than 14 days, and significantly reduced the expression of p53 and MMP-13 [129]. In addition, CAP- subcutaneous fat stromal cells derived exosomes (MSCs^SC^-Exos)/miR-199a-3p targeted chondrocytes by CAP peptide, electroporation loaded miR-199a-3p, inhibited mTOR pathway, enhanced autophagy activity, and further extended into the deep tissue of articular cartilage [130]. Targeted modification solves the problems of easy removal and poor targeting of Exos *in vivo*, and increases the local drug concentration.

③ The active ingredients of Exos were optimized by pretreatment of donor cells with cytokines. Interferon-γ-pretreated exosomes (iExos) pretreated with IFN-γ enriched Hsp70, the key anti-ageing molecule, maintained mitochondrial homeostasis, reduced ROS accumulation, and inhibited chondrocyte senescence by stabilizing AMPK-SIRT3 axis [131]. Cytokine pretreatment indirectly optimizes the function of Exos by ‘activating donor cells’. The process is simple and has good biocompatibility.

④ The anti-ageing activity of Exos was enhanced by culturing donor cells in hypoxic environment. Hypo-AD-MSCs-Exos were prepared by 1% O_2_ hypoxic culture, enriched 7 key mirnas including miR-381-3p and miR-122-5p, targeted regulation of oxidative stress and cell senescence pathways, and alleviate inflammatory senescence of chondrocytes, and the therapeutic effect is better than that of normoxia-derived Exos [118]. Hypo-BM-MSCs-Exos can significantly improve the anti-inflammatory, anti-ageing and analgesic multiple activities of Exos through hypoxic preconditioning, down-regulate the expression of p16, p21 and p53, and improve the subchondral bone structure [132]. Hypo-AD-MSCs-Exos target lumbar facet joints through tail vein injection, inhibit abnormal H-type blood vessel formation, reduce nociceptive nerve infiltration and relieve OA-related pain [133]. Hypoxic preconditioning can generally enhance the anti-ageing efficacy of Exos, which is a general functional optimization strategy.

⑤ There are also some relatively specific treatments that play an unexpected role in Exos: Through Alix-mediated miRNA sorting, strontium (Sr)-SMSC-EXOs pretreated with 100 nmol/L SRCl2 enriched miR-143-3p, targeted inhibition of Mfsd8, inhibited chondrocyte ferroptosis and delayed cartilage degeneration [134]. Ha-exos modified by HA can improve the targeted enrichment efficiency through the specific binding of HA to chondrocytes CD44, and HA itself has a synergistic effect of lubrifying joints and reducing inflammation [122]. This modification method has the advantages of functional enhancement and biocompatibility, and has outstanding potential for clinical transformation.

#### 2.4.3. Microvesicles

MVs derived from BM-MSCs can carry healthy mitochondria, which are endocytic and integrated into the mitochondrial network by OA chondrocytes. They promote mitochondrial biogenesis and restore mitochondrial membrane potential and ATP synthesis by activating PGC-1α/TFAM signalling pathway. It can reduce ROS accumulation and γH2AX mediated DNA damage, down-regulate senescence markers p16 and p21, and up-regulate the expression of anti-ageing proteins [98,135]. AD-MSCs-MVs can significantly up-regulate the expression of collagen II and aggrecan and down-regulate matrix decomposition enzymes such as MMP-13 and ADAMTS5 in OA chondrocytes by inhibiting the NF-κB inflammatory pathway and activating the SIRT1/FOXO3a pathway. To correct the ‘synthesis-catabolism imbalance’ [136,137]. Drug-loaded nanoparticles encapsulated in MVs (CD90^+^MVs) derived from synovial-derived CD90^+^MSCs could restart the chondrocyte cycle through FOXO signalling pathway, up-regulate cell cyclin-promoting factors such as cyclin B and PLK, reduce cell senescence caused by oxidative stress, and induce M2 polarization of macrophages. It can increase IL-10 secretion and inhibit joint inflammation [138]. In addition, MVs pre-stimulated by IFN-γ or TGF-β3, or loaded on ROS-responsive hydrogel or chitosan/gelatin composite carrier, can enhance anti-ageing activity and local retention, further improve the microstructure of OA subchondral bone, and delay the process of joint degeneration [59,137,139].

#### 2.4.4. Apoptotic bodies

Apoptotic bodies of AD-MSCs, especially Hypo-apoptotic extracellular vesicles (H-ApoEVs) after hypoxic preconditioning, can significantly promote the proliferation and chondrogenic differentiation of rat BM-MSCs, and up-regulate cartilage matrix synthesis markers such as SOX9, ACAN and COL II. It can down-regulate the expression of fibrocartilage gene COL I, and reduce the apoptosis rate and ageing-related phenotype of OA chondrocytes [140]. H-ApoEVs are rich in bioactive molecules such as miR-1246 and miR-210-3p. H-apoevs promote cell cycle progression by activating Wnt signalling pathway, inhibit chondrocyte apoptosis and senescence through PI3K-Akt/FoxO pathway, and enhance chondrogenic differentiation potential through TGF-β pathway. It can effectively improve the functional decline of OA-related cells [140]. Such apoptotic bodies can be phagocysed by macrophages through the phosphatidylserine ‘eat-me’ signal, inducing their polarization to anti-inflammatory M2 type, down-regulating pro-inflammatory factors such as IL-1β and TNF-α, up-regulating anti-inflammatory markers such as IL-10 and Arg, and reducing inflammation-mediated cell ageing damage [98,140]. In addition, apoptosome can also deliver functional mirnas, inhibit TLR3/COL10A1 and other pro-ageing pathways, restore the self-renewal ability of endogenous stem cells, and break the vicious cycle of ‘inflammation, aging, cartilage degradation’ [98].

#### 2.4.5. Secretions

In addition to the substances encapsulated in vesicles, soluble factor components secreted by MSC are also essential for the balance of cartilage matrix metabolism.

The secretomes (including soluble factors and EVs) of AD-MSCs can significantly up-regulate the expression of sox9 and acan in OA chondrocytes, and down-regulate the expression of matrix degrading enzymes (such as MMP-1 and MMP-13) and fibrosis marker col1a1 [141]. This secreome intervention can effectively correct the ‘synthesis-catabolism imbalance’ of chondrocytes and indirectly delay the decline of cartilage function [141]. MSC secretion is known to convert pro-inflammatory M1-like macrophages to an anti-inflammatory M2-like phenotype, reducing their secondary damage to joint tissue by inhibiting inflammatory pathways [45]. EVs derived from synovial fluid, which is rich in mitochondria, restored mitochondrial membrane potential and ATP synthesis, reduced ROS and γH2AX expression, down-regulated p16 and p21, and up-regulated anti-ageing protein SIRT1 by directly transferring mitochondria to OA chondrocytes [112]. BM-MSCs secretion can reduce chondrocyte inflammation and senescence and protect cartilage matrix synthesis by inhibiting the STING-NF-κB pathway [142].

Researchers have found that hPL pretreated UC-MSCs secrete IGF2 due to high GSH levels, which specifically rejuvenate the ageing phenotype of OA chondrocytes by activating autophagy pathway. Decreased expression of p16/p21 and positive rate of SA-β-galactosidase [88]. In addition, hUCMSCs secretome loaded on silk fibroin hydrogel achieved local sustained release, significantly improved the ageing state of BM-MSCs in aged rats, improved the ability of osteogenic differentiation, and reduced the expression of ageing markers [143].

#### 2.4.6. Conditioned media

CM is a mixture of stem cell culture supernatant, which contains soluble growth factors, cytokines and EVs mentioned above. It exerts anti-ageing effect through multi-component synergy, and its efficacy is significantly affected by the source of stem cells and culture conditions.

In addition, in the mouse model of femoral head necrosis, MSCs-CM treatment significantly reduced the number of senescent cells, reduced the level of SA-β-gal, and down-regulated the expression of p53, p21 and p16 [144]. iMSCs -CM containing HGF (hepatocyte growth factor), IL-1Rα, FGF2 and other active factors, reduced SA-β-gal positive rate and ROS production of OA chondrocytes and promoted 3D spheroid formation by anti-oxidation, promoting proliferation and down-regulating p21 expression [145]. Studies have found that the CM of regenerated BM-MSCs can improve the function of senescent cells [146]. In joints, young BM-MSCs-CM (P5) can improve the proliferation and osteogenic ability of aged MSC (P17), reduce the expression of SASP factors and prevent postmenopausal osteoporosis by inhibiting the p53 and p16^INK4a pathways. The mechanism may be migration to the regulation of senescence of OA cells [147]. In addition, injection of regenerated human MSCs-CM into the tail vein of mice can reduce the senescence of mouse BM-MSCs and related SASP, thereby inhibiting bone degradation [147]. Hypoxia-preconditioned BM-MSCs derived Hypo conditioned medium (HCM) is rich in growth factors such as Ang-1 and VEGF. It should be noted that EVs are the core active components, and high-dose EVs can replace the therapeutic effect of HCM. Inhibition of IL-1β-induced chondrocyte senescence and matrix degradation [104]. In cartilage ageing studies, AD-MSCs-CM can down-regulate IL-1β-induced SA-β-gal activity in chondrocytes, reduce oxidative stress and reduce the expression of p16 and p21 [68]. DPSCs-CM can reduce oxidative stress injury by up-regulating the expression of TIMP-1, improve the insufficient matrix synthesis of OA chondrocytes, and provide a new option for cell-free therapy [38]. UC-MSCs decellularization matrix (UC-MSCs -dECM) can inhibit the STING-NF-κB pathway, reduce the expression of inflammatory factors and matrix degrading enzymes, and reduce the expression of P16 and P21 to delay the ageing of chondrocytes [142].

The preparation process of CM is simple, can be produced on a large scale, and there is no risk related to cell transplantation [144]. Pretreatment with hypoxia and low serum can enhance the anti-ageing activity of CM, which provides an optimization direction for the preparation of clinical-grade products [104]. It can be combined with biological materials to construct a sustained release system to prolong the action time *in vivo* and improve the therapeutic effect [145]. These findings provide a solid foundation for the translation of MSC derivatives into clinical therapies against cellular senescence in OA.

### 2.5. Anti-ageing effects of MSCs and their derivatives on other OA cells

OA is regarded as a ‘whole joint’ disease, and the intervention of MSCs is not limited to cartilage. It has been reported that chondrocyte senescence alone cannot completely drive the pathological changes of OA [148]. Consistent with this, studies have found senescent cells in the synovium of OA patients [149]. Selective removal of these cells can reduce pain, promote cartilage development, and reduce the expression of ageing and inflammatory markers, thereby slowing the progression of OA [29,150,151].

Among them, FLSs from OA patients show obvious features of cellular senescence, Including increased expression of senescence marker p16INK4a, cell cycle changes, impaired autophagy and up-regulation of SASP levels compared with healthy controls [149,152,153]. Senescent FLSs highly express inflammatory cytokines, such as IL-1 and IL-6 [149], leading to a strong inflammatory response in the joint cavity [149]. Li et al. demonstrated that IL-1β treatment increased the expression of TNF-α and IL-6 in FLSs in an *in vitro model* of OA [119]. Furthermore, AD-MSCs co-culture significantly downregulated the expression of inflammatory-induced FLSs fibrosis markers (ACTA2, COL-I, COL-III), restored abnormal cell cycle distribution and reduced the proportion of SA-β-galactosidase positive cells. The mechanism is related to the inhibition of NF-κB, MAPK and other senescence-related pathways [69]. Moreover, IPFP-MSCs can not only significantly reduce cartilage degeneration and osteophytes formation in rabbit ACLT model, but also relieve subchondral bone sclerosis by intra-articular injection, and the source is convenient (discarded tissue during knee surgery) [154]. In addition, AD-MSCs-Exos intervention effectively alleviated the dysfunction caused by FLS ageing in OA by increasing the expression of miR-376c-3p, significantly reducing the expression of TNF-α and IL-6, and inhibiting the activation of WNT-β-catenin signalling pathway. Similarly, Ragni et al. isolated EVs secreted by AD-MSCs and found that they may regulate key pathways closely related to OA synovitis and reduce the expression of proinflammatory cytokines and chemokines in the FLS model [101]. Taken together, modulating the ageing process of FLSs may be an effective approach to treat OA. However, experimental co-culture of FLS from OA patients with MSCs showed a short-term down-regulation of inflammatory markers and no significant changes in long-term function, suggesting that future studies of stem cell therapy to alleviate FLS ageing should focus more on long-term effects [155].

In recent years, the role of subchondral bone in the pathogenesis of OA has attracted more and more attention. A recent study has shown that structural changes in subchondral bone are the main cause of OA in animal models [156], and early damage and bone loss of subchondral bone occur before cartilage degradation and osteophyte formation [156,157]. This is consistent with the clinical observation that OA is often accompanied by osteoporosis [158,159]. There is increasing evidence that bone remodelling in subchondral bone is associated with ageing of osteocytes and bone marrow-like cells, and SASP of these cells is associated with bone loss [160]. The balance between the number and function of osteoblasts and osteoclasts is one of the most important factors affecting bone remodelling in subchondral bone [161]. *In vitro* co-culture of UC-MSCs secretion with BM-MSCs from aged rats can ameliorate the ageing phenotype. Furthermore, the same secretion treated with hydrogel showed enhanced bone-forming capacity, restored stem cell potential and delayed age-related local bone loss in aged rats [143].

In addition, according to Yin et al. a new type of membrane-derived mesenchymal stem cells/stromal cells, amnion-derived mesenchymal stem cells/stromal cells (AMSCs), were able to effectively improve the symptoms of osteoporosis in mice by stimulating bone formation in osteoblasts and inhibiting bone resorption in osteoclasts [79]. In addition, AMSCs transplantation can inhibit oxidative stress and DNA damage in multiple organs of premature ageing mice, improve cell senescence and promote bone formation to rescue osteoporosis [162]. Some studies have further confirmed that AMSCs can improve mandibular osteoporosis in Bmi-1 deficient mice by down-regulating ageing related molecules such as p16, p21 and p53, increasing SOD1 activity, inhibiting bone resorption in osteoclasts and promoting bone formation in osteoblasts [32], which is consistent with the above conclusions. Interestingly, injection of tonsilla-derived mesenchymal stem cells/stromal cells (T-MSCs) into the tail vein of a mouse model of senile osteoporosis can effectively improve bone loss by regulating the function of preosteoblasts [163]. AD-MSCs derived CM, MVs and Exos significantly ameliorated IL-1β-induced senescence of OA osteoblasts, reduced SA-β-Gal activity and γH2AX foci number, and restored mitochondrial membrane potential [100]. For OA lesions, it has been found that MSCs and their derivatives can alleviate OA-related symptoms by reducing SA-β-Gal activity and γH2AX foci in senescent osteoblasts and improving mitochondrial function [100]. It is worth noting that the specific research on the use of MSCs and their derivatives to treat OA subchondral bone lesions is limited at present, which is an urgent direction for future in-depth research.

### 2.6. Anti-ageing effects of MSCs and their derivatives on OA joint microenvironment

It should be noted that MSCs not only target specific joint cells and exert a ‘one-to-one’ intervention effect, but also regulate the anti-ageing repair potential of the OA joint microenvironment more broadly. For example, intravenous injection of adMSCs can down-regulate systemic and joint local inflammatory factors (monocyte chemoattractant protein (MCP)-1 and TNF-α) through systemic immune regulation, improve gait abnormalities in multi-joint OA, and achieve ‘distal’ anti-ageing repair [71]. In addition, MSCs can promote the recovery of the concentration of HA and aggrecan-4 in the synovial fluid of the joint, improve the lubrication of the cartilage boundary from a biomechanical perspective, and protect chondrocytes from mechanical wear [60]. Autologous mFAT prepared by Lipogems and other technologies has been shown to continuously increase the content of proteoglycan in cartilage and improve the critical integrity of patients 24 months after a single injection in clinical follow-up, which is especially suitable for non-surgical regenerative therapy in patients with moderate to severe OA [66,164].

**Figure 1.**
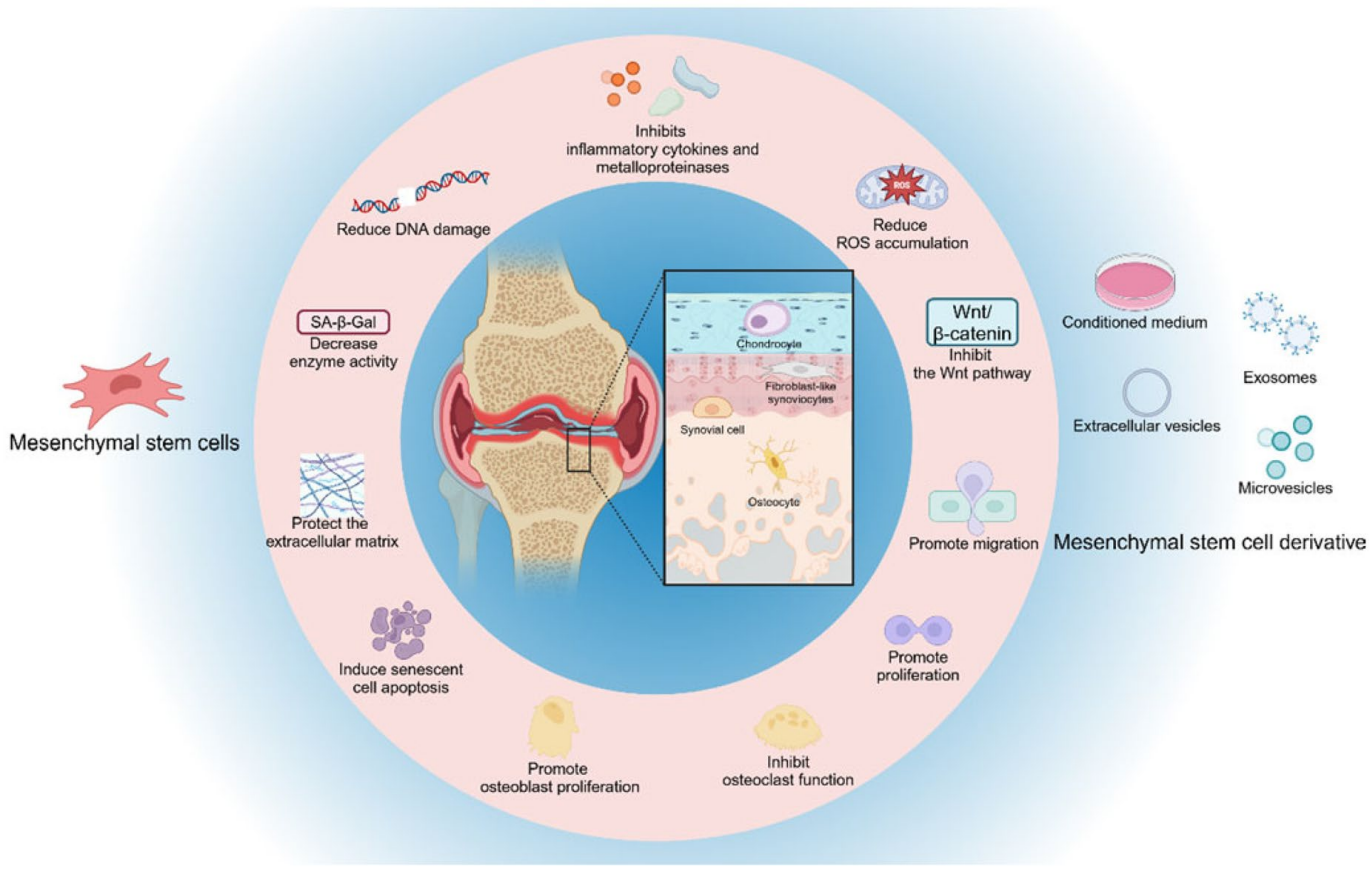
Anti-cell senescence effect of MSCs and their derivatives in OA. Mechanism of mesenchymal stem cells (MSCs) and their derivatives against cell senescence in the treatment of osteoarthritis (OA). MSCs regulate the aging progression of different cells in OA joints through multiple mechanisms (pink circle). ROS, reactive oxygen species; DNA, deoxyribonucleic acid.

The role of MSCs and their derivatives in the anti-ageing treatment of OA has been extensively studied, showing significant therapeutic potential (Figure 1). Further studies on the mechanisms and effects of these cells and their derivatives are expected to provide new insights and approaches for the treatment of OA.

### 2.7. Comparison of various types of MSCs and their derivatives

#### 2.7.1. Differences in MSCs derived from different sources

AD-MSCs are preferred for clinical transformation due to their convenient source (liposuction waste), long-lasting pain relief, significant functional improvement, low incidence of adverse events, no immune rejection in autologous transplantation, and low immunogenicity in allogeneic transplantation [97]. Ad-mscs can reduce the expression of ageing markers by inhibiting mTOR pathway and activating autophagy. It can reduce the expression of ageing markers by inhibiting mtor pathway and activating autophagy [57], and has the strongest potential for clinical transformation. BM-MSCs have outstanding early chondrogenic differentiation ability, which can improve mitochondrial function of chondrocytes and inhibit inflammation [52]. However, it is easy to senescence after long-term passage. Elderly donor EVs may promote the progression of OA due to high expression of SASP factor [64], and evs from young donors have better anti-ageing activity [106]. UC-MSCs has low ethical risk, very low immunogenicity, and no obvious immune response in allogeneic transplantation. Derivatives regulate oxidative stress and inhibit P53/P21 pathway by secreting SOD [55], which is more suitable for early and middle stage OA or allogeneic therapy. SMSCs have strong chondrogenic differentiation potential and targeted improvement of synovial fibroblast senescence [86], but invasive surgery is required to obtain them. IPFP-MSCs can be obtained from surgical waste tissue and improve subchondral bone sclerosis, synovitis and cartilage degeneration [77] with high safety. iMSCs can proliferate indefinitely, and the efficacy of Exos is better than that of some natural MSCs [120], but there is batch heterogeneity [107]. Most MSCs preferentially target senescent chondrocytes and synovial cells, and have no off-target effect on healthy cells [88]. The anti-ageing activity of young donors is generally better than that of old donors [106].

#### 2.7.2. Differences related to different types of stem cell derivatives

Exos are the first choice of cell-free therapy, with long half-life *in vivo* (up to 6.774 days after engineered modification), high cartilage uptake rate, precise delivery of miRNA (such as miR-376c-3p) regulatory pathways [119], no risk of tumourigenesis and embolism, and minimal immunogenicity. After large-scale preparation, the batch consistency is good [101]. EVs contain subsets such as Exos and MVs, which can transfer mitochondria and regulate inflammation [59], but the stability is lower than that of Exos [102]. CM is a mixture of soluble factors and EVs, and EVs are the core active ingredients [104], which can synergistically anti-inflammatory and anti-ageing, but with a short half-life and requires sustained release of carriers [100]. Secretome focuses on the effect of soluble factors. After pretreatment, the active ingredients can be optimized to specifically rejuvenate the ageing phenotype of OA chondrocytes [88]. All derivatives have no serious adverse events, and Exos and EVs have stronger targeting and better clinical transformation potential [99].

#### 2.7.3. Differences related to different treatment modalities

Local injection (intra-articular injection) is the mainstream method, which directly affects the lesion site, has high drug concentration and few side effects, and is suitable for early and middle OA [66]. It can deliver MSC, Exos, etc., quickly relieve inflammation and improve cartilage damage [45]. Combined with hydrogel, it can achieve CM and secretion sustained release [85]. Intravenous injection is suitable for patients with multi-joint OA or systemic inflammation. Intravenous injection of adMSCs can systematically down-regulate inflammatory factors [71], but the targeting is weak, and MSCs are easy to be retained in the lung, while intravenous injection of EVs is safer [45]. Surgical transplantation (composite scaffold transplantation) is suitable for severe cartilage defects. 3D bioprinting and collagen scaffold loaded MSC can achieve cartilagen-bone integrated repair [93], but the trauma is large and the operation is complex. Combined treatment (such as acupotomology combined with AD-MSCs, HTO combined with AD-MSCs) can synergistically improve the mechanical environment and repair ability, and the effect is better than that of single method [96]. Local injection is more likely to target senescent cells in the joint, intravenous injection focuses on systemic inflammation regulation, and surgical transplantation directly acts on the defect site [87]. Local injection combined with vector loading is a promising combination with outstanding potential for clinical transformation [66].

## 3. Future potential and challenges

The clinical translation of MSCs and their metabolites in the treatment of OA by anti-cellular senescence still faces core challenges and breakthroughs. In terms of long-term safety, existing studies have confirmed that MSCs, Exos, EVs and other derivatives have no abnormal proliferation performance *in vitro* culture, and no signs of tumour formation have been observed *in vivo* experiments [165,166]. The risk of ectopic tissue formation can be effectively avoided by targeted cartilage modification techniques such as 3D printing combined with CAP peptide [167,168]. However, there is currently a lack of long-term follow-up data of more than 1 year in large animals such as pigs and monkeys, which cannot fully verify the histocompatibility and functional stability in long-term application [113,132]. The regulatory obstacles are also prominent. As the core derivatives, the methods for particle size detection, surface marker identification and functional component analysis of Exos have not yet formed a unified standard [105,169], which makes it difficult to compare the results of different studies [105,169]. At the level of quality control, Exos derived from iMSCs -Exos have the problem of batch heterogeneity [107,170]. Strict quality control process should be established in the large-scale production process to ensure the consistency of product activity and safety [76]. The efficacy of MSCs is significantly affected by factors such as donor age and health status. EVs from young donors have stronger anti-ageing activity, and donor MSCs with high expression of IDO/TSG6 have better therapeutic effect [75,106]. This heterogeneity increases the uncertainty of clinical application [75,106]. This heterogeneity increases the uncertainty of clinical application. There is an urgent need to develop a ‘donor screening scoring system’ to screen high-quality donors through quantitative indicators

From the perspective of sub-dimensions, the risk of immune rejection is the key consideration for the clinical application of MSCs. Due to the consistent genetic background with the recipient, autologous MSCs can completely avoid immune rejection and are suitable for long-term treatment [61,66]. Allogeneic UC-MSCs have low expression of HLA-DR molecules, no obvious inflammatory reaction after allogeneic transplantation, and good biocompatibility [75]. In order to further reduce the immunogenicity, autologous or UC-MSCs can be preferred in clinical practice, or EVs/Exos can be modified by HA modification and other engineering methods to reduce immune recognition [122]. The standardization problem needs a collaborative breakthrough from multiple links. In the process of isolation and expansion, the use of serum-free culture system can reduce the contamination of heterogeneous proteins, and the uniform passage times (P3-P5) can ensure the activity and stemness of MSCs [76]. In terms of donor screening, it is necessary to establish a comprehensive evaluation system including cell activity, marker expression, anti-ageing factor secretion level and other indicators, and preferentially select donors with high expression of IDO/TSG6 [75]. By integrating the process of ‘donor quality control + serum-free culture + standardized passage’, product heterogeneity can be effectively reduced [91]. Optimization of the delivery method is the core to improve the efficacy. Although traditional intra-articular injection is minimally invasive, the half-life of MSCs and derivatives *in vivo* is short (1–2 days) [113], which is difficult to maintain long-term therapeutic effect [113]. The hydrogel loading technology can prolong the retention time of preparations in the joint to more than 14 days. Some ROS-sensitive hydrogels can also realize intelligent response release to adapt to the local high inflammatory microenvironment of OA [59,129]. 3D printing technology can accurately match the morphology of cartilage defects, realize the sustained-release delivery of EVs/Exos, and provide a bionic growth microenvironment for cells [93,171]. In general, the scheme of hydrogels loaded with engineered Exos (such as CAP-EXO-HA) takes into account both targeting and long-term effect, which is the most potential delivery strategy at present [129].

Future research directions will focus on efficiency improvement, scenario expansion, and mechanism deepening. Engineering modification and upgrading will further strengthen the precision of treatment. Through the multi-target loading strategy, EVs/Exos can simultaneously load functional molecules with synergistic effects such as miR-140-5p and miR-27b-3p, which can strengthen the regulation of OA-related ageing pathways [114,130]. The combination of targeted peptides can improve the recognition specificity of the preparation on chondrocytes and reduce off-target effects. Combined therapy is an important direction to expand the treatment scenario. The combination of EVs/Exos and senescolytic drugs can achieve the synergistic effect of ‘clearing senescent cells + repairing cartilage damage’ [58]. Combined with mechanical corrective surgery, it can improve the joint mechanical environment and cellular repair microenvironment at the same time, and improve the therapeutic effect of moderate to severe OA [74]. Clinical transformation needs to focus on patients with moderate to severe OA, carry out large-sample and multi-center clinical trials, optimize the dosage and cycle of drug administration [113,118], and clarify the suitable scheme for OA in different pathological stages [113,118]. Further mechanistic studies need to identify core functional molecules in derivatives, such as Hsp70 in Exos [131], miR-221-3p [126], etc., to provide a basis for the development of ‘cell-free and vesicle free’ targeted agents, and promote OA treatment from ‘cell/vesicle therapy’ to ‘precision molecular therapy’ [126,131]. To provide safer and more efficient treatment options for patients.

## 4. Conclusion

Current research indicates that MSCs and their derivatives exert anti-senescence effects through various mechanisms and show great potential for the treatment of OA. To date, MSCs can effectively delay or reverse the pathological progression of OA mainly by modulating senescence-related signalling pathways, reducing the secretion of pro-inflammatory factors and repairing damaged cartilage. However, despite these encouraging results, the specific roles of MSCs and their derivatives at the molecular level and in clinical applications require further exploration. Future research should focus on the underlying mechanisms of MSCs-mediated anti-senescence effects and conduct large-scale clinical trials to verify their actual efficacy and safety in OA treatment. This will be a key direction in future OA research and is expected to provide new breakthroughs in the treatment of this disease.

## Data Availability

Data sharing is not applicable to this article as no data were created or analyzed in this study.
